# The relation of individual and collective narcissism and belief in COVID-19 conspiracy theories: the moderating effects of need for uniqueness and belonging

**DOI:** 10.1007/s44202-022-00047-1

**Published:** 2022-08-22

**Authors:** Bengi Ük, Hasan G Bahcekapili

**Affiliations:** Department of Psychology, Istanbul Medipol University, Göztepe, Bodrum Kat, Anadolu Hisarı Mah. Kavacık Kavşağı No:19 D2, D:Blok 1, Beykoz, 34810 Istanbul, Turkey

**Keywords:** Covid-19, Conspiracy, Grandiose narcissism, Uniqueness, Collective narcissism

## Abstract

The Covid-19 pandemic not only led to chaos and uncertainty, it also brought about many conspiracy theories. In the last two decades, with considerable amount of research, social psychologists have begun to unravel the personality traits underlying conspiracy theories. One such trait is narcissism where the need to distinguish oneself from others might be satisfied by holding beliefs that are different from the general population. In this research, we focus, for the first time in the literature, on both collective and grandiose narcissism’s predictive effects on Covid-19 conspiracy theories and the possible moderation of need for uniqueness (feeling oneself to be special and different from others) and belonging (feeling oneself to be part of a larger and worthy whole). In a Turkish sample (*N* = 309), we found that both collective and grandiose narcissism were significant predictors of Covid-19 conspiracy. In addition, when the need to feel special was high, grandiose narcissists, but not collective narcissists, tended to believe in Covid-19 conspiracies. Finally, we found that generic conspiracy beliefs were also important predictors of Covid-19 conspiracy theories. Our research illuminates the link between narcissism and Covid-19 conspiracy theories. Future research should look for other possible moderating factors between collective narcissism and conspiracy beliefs in the context of Covid-19.

## Introduction

Conspiracy theories try to explain important events and situations with the maleficent acts of some secret and powerful group or people [14]. These ‘malicious’ people or groups can be the government, foreign countries, certain racial/ethnic origins such as Jews or independent groups such as pharmaceutical industries [38]. Although findings in the literature revealed that conspiracy theories have become widespread with the ease of information sharing in the age of technology [9], these theories have existed in almost every period in human history when fear and anxiety were dominant (e.g., [12, 37]). In a study conducted in 2004, the number of people who believed that the American government was involved in the September 11 attacks was more than 15% [41]. Gallup’s research in 2019 found that up to 68% of the American public believed that the government had some important information about UFOs but kept this information from the public. The present study attempts to understand belief in conspiracy theories in terms of the satisfaction of some personal needs.

Studies have shown that conspiracy theories are common in many regions including Asia, Africa, and the Middle East, suggesting that belief in conspiracy theories is part of human nature and may meet certain needs (e.g. [6, 38]). According to Douglas et al. [14], it is possible to classify the social needs met by conspiracy theories in three categories: epistemic, existential, and social. Epistemic motivation refers to the need to reduce uncertainty in the environment and see meaningful patterns. Studies on this subject have indeed shown that individuals with hyperactive agency detection are more prone to conspiracy theories (e.g., [10, 13]). Secondly, existential motivation refers to the need to feel secure by gaining control. As shown in the literature, when people believe that the situation is beyond their control and feel anxious, they are more likely to believe in conspiracy theories [23]. Finally, social motivation refers to the need to hold on to a positive image of oneself and the group one belongs to. Since conspiracy theories usually involve the hidden ambitions and plans of a powerful and malicious outgroup, people tend to see themselves and the group they belong to as having the moral high ground through these theories [8].

Although meeting these three basic motivations may explain to some extent why people have conspiracy beliefs, other research sought to identify more specific individual traits related to belief in conspiracy theories. Some studies examined the relationship between conspiratorial thinking to mental processes and found a close relationship between low analytic thinking and belief in conspiracy theories [2, 34]. Religious and paranormal beliefs were also associated with conspiracy beliefs [10]. In addition, several studies looking at which personality traits increase conspiracy theories have found that schizotypal [24] and paranoid [6] traits were linked to these theories.

Another important personality trait closely related to belief in conspiracy theories is narcissism. In many studies, it was shown that people with grandiose narcissistic characteristics have higher levels of belief in conspiracy theories [8]. The definition of grandiose narcissism includes features such as grandiosity, exhibitionism, entitlement, arrogance, desire for attention, excessive demandingness, and inability to see the needs of others [3]. The need for superiority has been frequently discussed in the literature, and it has been stated that people who display these characteristics attribute negative characteristics to the outside to keep their selves intact [1]. At this point, it is thought that belief in “persons/groups with maleficent and secret purposes” is related to the tendency to develop a purely positive perception of oneself by attributing negative characteristics to others. Cichocka et al. [8] explained this situation with the tendency of narcissistic people to perceive the behaviors of others as against themselves and therefore their tendency toward paranoid thoughts.

Another possible link between belief in conspiracy theories and narcissistic personality traits is narcissistic people’s need to be unique and different. The need for uniqueness is defined as a person’s need to feel different and unique among others, and it can emerge as both a lasting and a temporary feature [29]. In this context, it is seen that individuals with narcissistic characteristics need to see themselves as different, especially in situations where people act en masse and are perceived to be very similar to each other. As shown in the literature, one of the underlying reasons for conspiracy theories is the need to be unique (e.g., [25, 27]). It has been shown in previous studies that the need to be unique leads to belief in conspiracy theories [21]. Considering that conspiracy theories often emerge in environments where anxiety and chaos prevail and are considered as an alternative to official accounts, people think that they have some information through conspiracy theories that others are unaware, that they are enlightened when others are in the dark, and they feel special [12]. In addition, in this way, people can attract the attention of the public with alternative explanations and satisfy their narcissistic needs while being the focus of attention [13].

Collective narcissism, on the other hand, is defined as the belief that one’s group is exceptional and deserves exceptional treatment but is not sufficiently recognized by others [19]. In this context, collective narcissism can be seen as a reflection of grandiose narcissism on a social group basis [18]. In collective narcissism, the idealized person is not the person himself but the group to which he belongs, and the person strengthens his self-esteem by basing it on his belief that the group he belongs to is privileged [20]. Parallel to the attribution of negative features by individuals with individual narcissistic characteristics, exaggerated positive perception of the group they belong to can be reflected in collective narcissists as prejudice against other groups in forms such as racism or sexism [15]. In addition, there is an exaggerated perception of threat in collective narcissists to the group they belong to and there is an extra sensitivity in this regard. For this reason, studies in the literature have shown that in uncertain or anxiety provoking situations, people who have collective narcissistic characteristics tend to see these situations as threats to their national image and attribute them to external forces [21].

Since originating in China and spreading all around the world, as of January 2022, the Covid-19 pandemic reached over 296 million cases and about 5 million deaths [40]. With this mass pandemic, it is seen that people all over the world exhibit similar fears, beliefs and attitudes. During this period, conspiracy theories took many different forms and targeted different groups. Some of the most common ones address the emergence of the virus, while others focus on prevention and treatments [36]. However, they all share the belief that some groups or individuals who have ‘ulterior motives’ take part in this epidemic. In a pandemic where uncertainty is so intense, conspiracy theories present a roadmap for people about what they should believe, how they should behave and what they should avoid [30]. Gligorić et al. [16] examined the three needs (epistemic, existential and social) discussed by Douglas et al. [14] in the context of Covid-19. First, they found that people with an intolerance of uncertainty believe more in conspiracy theories about the Covid-19 outbreak. Secondly, they found that the more people want to have power and control over the events around them, or they think that control is out of their hands, the more they tend toward conspiracy theories about the Covid-19 epidemic. Finally, they looked at the effect of people’s desire to develop a positive attitude towards themselves and the group they belong to and found that grandiose narcissism and collective narcissism are the most important factors in believing in conspiracies about Covid-19.

Although both individual grandiosity and collective narcissism increase the belief in conspiracy theories about the Covid-19 epidemic, the connection of these two features with conspiracy theories is different. First, the need for ‘uniqueness’ in people with grandiose narcissism is thought to be an important tool in believing in conspiracy theories about Covid-19. As shown in the literature, people who need to be different believe in conspiracy theories and define themselves as ‘seeing and knowing ulterior motives that others do not see’ and feel special [27]. On the other hand, the need of people with collective narcissism is to “belong” and to strengthen their self-esteem through the group in which they feel this sense of belonging [20]. Sternisko et al. [32] showed that people who exhibit collective narcissistic characteristics during the Covid-19 epidemic tend to see this epidemic as a threat to their ideal national identity, and therefore believe in conspiracy theories. They also demonstrated that belief in conspiracies led to less support for COVID-related public health policies. Another difference between individual narcissism and collective narcissism regarding conspiracy theories is the differences in the conspiracy theories they believe in. As shown in previous research, individual grandiose narcissism predicts conspiracy theories regardless of whether the conspiracy theories are ‘of the group one belongs to' or 'of an outgroup’, while collective narcissism only predicts conspiracy theories about the outgroup [8]. Cichocka et al. [8] attribute this difference to the fact that what individuals with individual narcissistic characteristics want to protect is their self-perception rather than their group perception.

The aim of the current study is to examine the relationship of both individual grandiosity and collective narcissism with the conspiracy theories put forward during the Covid-19 epidemic and the possible moderating effects of need for uniqueness and belonging. In line with the previous findings, both features are expected to significantly predict the widely spread conspiracies about Covid-19. One main prediction of the study is that the conspiracy theories believed by people with collective narcissistic characteristics include malicious out-groups (e.g., “The coronavirus was produced in laboratories in China and was deliberately released to the public”). To test this prediction, we measured the degree of belonging to one’s national identity with a one-item ‘belongingness’ question. A second prediction is that individuals with grandiose individual narcissistic characteristics are prone to conspiracy theories targeting external or internal groups (for example, the state or the media), since the motivation for believing in conspiracy theories will be ‘feeling different’. To test this prediction, we used the need for uniqueness scale. In addition, as shown in the literature, people who believe in a conspiracy theory are more likely to believe in other conspiracy theories, even if they contradict each other [17]. Therefore, in the present study, the relationship between belief in general conspiracy theories and belief in conspiracy theories about Covid-19 is also examined. Designing two separate moderations analyses, we expected that both individual and collective narcissism would predict Covid-19 theories with the moderating effects of need for uniqueness and belonging.

## Method

### Participants

In the first part of the study, we aimed to reach psychology students in exchange of extra credit. The survey was completed by 309 undergraduate students (271 women), aged 18–44 (M = 21.68, SD = 3.10). All participants were native Turkish speakers and all materials were presented in Turkish.

### Procedure

Through an online questionnaire, the participants responded to measures of belonging, collective narcissism, personal sense of uniqueness, short Dark Triad’s narcissism, generic conspiracist beliefs and beliefs in Covid-19 conspiracy theories. At the end of study, we gave the participants a cluster of sociodemographic questions including age, gender, political ideology (1 = extreme left, 7 = extreme right) and religiosity (1 = not religious at all, 7 = very religious). The whole process lasted about 15–20 min.

### Measures

#### Belonging

First, participants were asked two questions about their nationality. If they described themselves as Turkish, they were asked to rate the extent of their belonging to Turkish identity between 1 and 7. While 1 means ‘I don’t feel related at all’, 7 means ‘I feel completely related’. Most participants identified themselves as Turkish (n = 282). Other common nationalities were Kurdish (n = 11) and all the rest (n = 7). For people who identified themselves with other nationalities, belongingness item and collective narcissism scale were not given.

#### Collective Narcissism Scale (CNS)

The Collective Narcissism Scale was developed by Zavala et al. [19] with nine items on a scale from 1 = strongly disagree to 7 = strongly agree. The scale was found reliable with a Cronbach Alpha of 0.83. The scale aims to assess the belief that one’s own in-group is exceptional but not sufficiently recognized by others. Participants were asked to what extent they agreed with statements such as “If my group had a major say in the world, the world would be a much better place”. Turkish adaptation of the scale was done by Zavala and her colleagues [22]. In the current study, the in-group was chosen as ‘being Turkish’ and the items were phrased accordingly. The 282 participants who described themselves as ‘Turkish’ were given the CNS.

#### Personal Sense of Uniqueness Scale (PSU)

The Personal Sense of Uniqueness Scale (PSU) was developed by Şimşek and Yalınçetin [35] as a 5-item, 5-point scale (1 = strongly disagree to 5 = strongly agree) for the Turkish population It aims to measure the individual’s sense of uniqueness with items like “As people get to know me more, they begin to recognize my special features”. Its test–retest reliability was found .80 and Cronbach Alpha as .81.

#### Short Dark Triad-Narcissism

The short version of the Dark Triad Scale was developed by Jones and Paulhus [26]. It has 27 items measuring psychopathy, Machiavellism and narcissism. In the current study, we used only the narcissism subscale with nine items ranging from 1 (strongly disagree) to 5 (strongly agree). The Turkish version of the scale [31] has good reliability (α = .79). A sample item is “I insist on getting the respect I deserve”.

#### Generic Conspiracist Beliefs

Generic Conspiracist Beliefs is a 15-item scale developed by Brotherton et al. [7]. It aims to measure the general tendency to believe common conspiracy theories. A sample item is “A lot of important information is deliberately concealed from the public out of self-interest”. The reliability of the Turkish translation is very good (α = .90; [2]).

#### Belief in COVID-19 conspiracy theories

To estimate people’s beliefs about conspiracy theories regarding Covid-19, we developed an 11-item scale. The participants rated how much they agreed with statements in two main categories. One category is in-group conspiracy theories, the other is out-group theories. All five of the out-group conspiracy items were translated from other studies (e.g., [5]) such as “I think that the development of the epidemic may be beneficial to certain groups of whose interests we are unaware”. To determine the in-group items for the Turkish population, social media sites were checked, the most common theories were collected and gathered into six items such as “I think that the government uses the pandemic as a pretext to limit the rights and freedoms of the citizens”. In this research, item analysis was done to determine the reliability of the scale, which was α = .86.

## Results

### Zero-order correlations

First, normality of the variables was tested. All variables were normally distributed. Pearson correlation analysis was used to see all the correlations between variables as displayed in Table 1. Overall, Covid-19 conspiracy beliefs had the strongest correlation with general conspiracy beliefs with *r* = .69, *p* < .001. In addition, collective narcissism (*r* = .44, *p* < .001), narcissism (*r* = .32, *p* < .001), belonging (*r* = .17, *p* < .05) and personal sense of uniqueness (*r* = .16, *p* < .05) were significantly correlated with Covid-19 conspiracy beliefs. In addition, as expected, all other variables had positive significant correlations with each other. When Covid-19 conspiracy beliefs were categorized as ingroup and outgroup, collective narcissism had a stronger correlation with outgroup conspiracy theories (*r* = .46, *p* < .001) compared to ingroup ones (*r* = .34, *p* < .001). This difference was marginally significant, *z* (282) = 1.69, *p* = .09. On the other hand, for individual narcissism, outgroup and ingroup conspiracy theories did not differ; *r* = .30, *p* < .001 and *r* = .28, *p* < .001, respectively.

**Table 1:** Bivariate correlations between variables

Variable name	1	2	3	4	5	6	7	8
1. CO19C								
2. GCB	.690**							
3. O-CO19C	.904**	.670**						
4. I-CO19C	.918**	.590**	.660**					
5. CNS	.438**	.364**	.462**	.343**				
6. Belonging	.175**	.184**	.228**	.095	.546**			
7. PSU	.159**	.171**	.126*	.163**	.299**	.194**		
8. SDT-N	.323**	.315**	.307**	.283**	.351**	.224**	.355**	

^**^p < .01, *p < *.05,* CO19C: Belief in COVID19 Conspiracy Theories, GCB: Generic Conspiracy Beliefs, O-CO19C: Belief in Outgroup COVID19 Conspiracy Theories, I-CO19C: Belief in Ingroup COVID19 Conspiracy Theories, CNS: Collective Narcissism Scale, PSU: Personal Sense of Uniqueness Scale, SDT-N: Short Dark Triad-Narcissism Scale

### Exploratory analysis

Hierarchical regression analyses were conducted to see the effects of two narcissism types, sociodemographic variables, and generic conspiracy beliefs on Covid-19 conspiracy theories. In the first step, we entered political ideology and religiosity due to their significant correlations with Covid-19 conspiracy theories. Together they explained only 4% of the variation, *F* (2, 279) = 6.25, *p* = .002. In the second block analysis, we added the two narcissism types. It increased the variance explained to 22%, *F* (4, 277) = 20.03, *p* < .001. In the final step, generic conspiracist beliefs was added into model. They added 29% to the explained variation in Covid-19 conspiracy theories and raised total variance to 51% *F* (5, 276) = 57.04, *p* < .000. Among all variables, generic conspiracist beliefs was by far the most important predictor.

### Moderation analyses

#### Belonging, collective narcissism and ‘Outgroup’ Covid-19 conspiracy theories

The PROCESS Macro (model 1) was used to test the moderating effect of belonging on the relationship between collective narcissism and belief in Covid-19 conspiracy theories. The results are presented in Table 2.

**Table 2:** The moderating effect of belonging on the relationship between collective narcissism and outgroup COVID19 conspiracy theories and covariates of general conspiracy beliefs, religiosity, and ideology

	B	se	t	p	LLCI	ULCI
Constant	− 3.40	2.32	− 1.46	.143	− 7.96	1.16
CNI (A)	.058	.069	.836	.403	− .078	.194
Belonging (B)	− .68	.467	− 1.46	.145	− 1.60	.237
A × B	.011	.013	.897	.370	− .013	.037
Religiosity	.321	.156	2.05	.040	.013	.629
Ideology	.515	.198	2.60	.009	.125	.905
GCB	.230	.018	12.51	.000	.194	.266

B: Beta, SE: standard error, p: p value (probability value) LLCI: lower limit confidence interval, ULCI: upper limit confidence interval

For the overall model, *R*^2^ value of .21 revealed that the predictors explained 21% of the variance in the outcome with *F* (3, 278) = 25.44, *p* < .001. First, collective narcissism had a positive effect on outgroup Covid-19 conspiracy theories (*b* = .203, t (278) = 2.34, *p* < .05). Belonging did not have a significant effect, *p* > .05. In addition, the interaction term between collective narcissism and belonging did not have a significant effect on outgroup Covid-19 conspiracy theories. This result indicates that while collective narcissism is a predictor of believing outgroup Covid-19 conspiracies, belonging does not moderate the effect.

In addition, because the three variables had high significant correlations with Covid-19 conspiracies, we added belief in general conspiracy theories, religiosity and ideology as covariates and designed the model again. In this model, *R*^2^ value rose to .52, which means that the predictors explained 52% variance in the outcome with *F* (6, 275) = 49.14, *p* < .001. However, when we added the covariates, neither collective narcissism nor belonging was significant predictors of outgroup Covid-19 conspiracy theories. The interaction between them was non-significant. Instead, all three covariates had positive effects on outgroup Covid-19 conspiracy theories. Firstly, belief in general conspiracy theories had a significant positive effect, *b* = .23, t (275) = 12.52, *p* < .001. In addition, as religiosity increased, belief in outgroup Covid-19 conspiracies increased, *b* = .32, t (275) = 2.05, *p* < .05. Finally, as right-wing ideology increased, outgroup Covid-19 conspiracies increased too, *b* = .51, t (275) = 2.6, *p* < .01.

### Uniqueness, narcissism and Covid-19 conspiracy theories

Another PROCESS Macro (model 1) was used to verify the moderating effect of uniqueness on the relationship between narcissism and belief in Covid-19 conspiracy theories. The results are presented in Table 3.

**Table 3:** The moderating effect of uniqueness on the relationship between narcissism and COVID19 conspiracy theories and covariates of general conspiracy beliefs, religiosity and ideology

	B	se	t	p	LLCI	ULCI
Constant	29.04	15.23	1.90	.057	− .927	59.01
SDT-N (A)	− 1.20	.554	− 2.17	.030	− 2.29	− .113
PSU (B)	− 2.16	.887	− 2.44	.015	− 3.91	− .421
A × B	.081	.032	2.55	.011	.018	.144
Religiosity	.592	.272	2.17	.030	.056	1.12
Ideology	.507	.361	1.40	.162	− .204	1.21
GCB	.495	.032	15.17	.000	.430	.559

B: Beta, SE: standard error, p: p value (probability value) LLCI: lower limit confidence interval, ULCI: upper limit confidence interval

For the overall model, *R*^2^ value of .12 revealed that the predictors explained 12% variance in the outcome, *F* (3, 305) = 13.97, *p* < .001. First, narcissism did not have a significant effect on Covid-19 conspiracy theories; *p* > .05. However, uniqueness had a negative effect: as uniqueness increased, belief in Covid-19 conspiracy theories decreased (*b* = − 2.43, t(305) = − 2.04, *p* < .05). Thirdly, interaction between the two had a positive effect on Covid-19 conspiracy theories, *b* = .09, t (305) = 2.20, *p* < .05).

In addition, because these three variables had high significant correlations with Covid-19 conspiracies, we added belief in general conspiracy theories, religiosity and ideology as covariates and designed the model again. In this model, *R*^2^ value rose to .52, *F* (6, 302) = 54.4, *p* < .001. In this model, narcissism showed a negative significant effect on Covid-19 conspiracy theories, *b* = − 1.20, t (302) = − 2.17, *p* < .05. Uniqueness had a significant negative effect, *b* = − 2.16, t (302) = − 2.44, *p* < .05. The interaction term between narcissism and uniqueness had a positive significant effect, *b* = .08, t (302) = 2.55, *p* < .05. In addition, two of the covariates, belief in general conspiracy theories and religiosity, had positive significant effects on Covid-19 conspiracy beliefs. For general conspiracy beliefs, *b* = .49, t (302) = 15.17, *p* < .001. Additionally, or religiosity, *b* = .59, t (302) = 2.17, *p* < .05. Ideology did not show any significant effect on Covid-19 conspiracy theories; *p* > .05.

The addition of the interaction term produced a significant change in the model, *F* (1, 302) = 6.5, *p* < .05, *R*^2^ change is 0.01. For low and average uniqueness (− 1SD below the mean and mean), narcissism did not predict Covid-19 conspiracy theories. However, for + 1SD above the mean, *b* = .42, t (302) = 3.17, *p* < .05, which means that for high uniqueness scores, narcissism positively predicted Covid-19 conspiracy theories. The results are presented in Table 4.

**Table 4:** Effects of narcissism at values of uniqueness

Uniqueness	Effect	SE	t	p	LLCI	ULCI
− 1SD	− .035	.130	− .272	.785	− .292	.221
M	.176	.096	1.82	.069	− .014	.366
+ 1SD	.388	.124	3.11	.002	.143	.633

## Discussion

The aim of this study was to explore the role of two forms of narcissism in the endorsement of Covid-19 conspiracies. We tested the relationship of conspiracy beliefs and both types of narcissism (collective and grandiose) with the moderating effects of need for uniqueness and belonging. The results mostly supported our predictions. First, as expected, significant positive correlations were found between generic conspiracy beliefs, Covid-19 conspiracies, collective narcissism, and grandiose narcissism. Personal sense of uniqueness was also found to be significantly correlated with all these variables. In addition, belonging to Turkish identity was found to be significantly correlated with all variables except in-group Covid-19 conspiracies, which was in line with our expectations.

As regards sociodemographic variables, both religiosity and ideology had significant correlations with Covid-19 conspiracy theories. First, people with right-wing ideology had a higher tendency to believe in outgroup Covid-19 conspiracies. This relationship was not seen for ingroup conspiracies. In addition, people who reported themselves as more religious tended to believe in both types of Covid-19 conspiracies. It was previously shown that these two concepts are related (e.g., [2, 10]). A possible explanation for this relationship is hyperactive agency detection. Agency detection is a natural phenomenon which can be seen in both animals and humans and defined as automatically inferring the presence of an intelligent agent behind events that are unlikely to be purely mechanically caused. However, as Barrett [4] argued, many people may have an overly sensitive agency detection mechanism. As a by-product of their tendency to quickly detect and react to an event which is caused by an agent, people sometimes attribute intention and agency to natural events and see them as consequences of hidden purposes [4]. As Douglas and her colleagues [13] showed, people who inanimate objects and attribute intention to natural events tend to be more religious and believe in conspiracy theories. Besides, as Gligorić et al. [16] showed in his research, paranormal beliefs can be explained in the same way. Future research may further elucidate the involvement of hypersensitive agency detection in paranormal, religious, and conspiratorial beliefs.

Collective and grandiose narcissism significantly predicted Covid-19 conspiracies. When we added generic conspiracy beliefs, religiosity, and ideology, predictive power increased dramatically. In fact, the most significant predictor of Covid-19 conspiracies was generic conspiracy beliefs. This result is also consistent with previous findings (e.g., [11, 17]) which exemplify the so-called “monological belief system”: that believing in one conspiracy theory makes it more likely to believe in other conspiracies. These studies found that belief in a particular conspiracy theory is a significant predictor of believing in other conspiracy theories even when these conspiracies are unrelated [11] or even contradictory [39]. In line with the literature, we found that all items in the Covid-19 conspiracy scale were significantly correlated. For example, two items which were correlated are “The media is trying to make the state look bad by exaggerating the coronavirus disease” and “The government aims to scare the public by showing the number of patients higher than it actually is”. Although they seem contradictory, people who believe in one tend to believe in the other.

We next examined the role of the two types of narcissism on Covid-19 conspiracy beliefs to reveal possible moderating effects. First, we looked at the moderating effect of ‘belonging’ in collective narcissism and Covid-19 conspiracies. When Covid-19 conspiracies were separated as outgroup and ingroup, collective narcissism positively predicted outgroup conspiracies. In addition, it had a smaller effect on ingroup conspiracies, which was an unexpected result. In light of the previous research, we expected that people who have high national narcissism would be susceptible to conspiracies only when their national image was under threat [32]. Indeed, they have higher conspiracy beliefs about ‘others’. However, they also believe ingroup conspiracies to some extent. This result may be due to the nature of our scale. The ingroup subscale involve items about the Turkish society including the media, medical companies and government. It is possible that some of these items were not seen by our participants as representative of their national identity. We also expected a significant moderating effect of belonging to Turkish identity between collective narcissism and outgroup Covid-19 conspiracies. Although ‘belonging’ had a strong positive correlation with collective narcissism and a mild positive correlation with outgroup Covid-19 conspiracies, it did not moderate their relationship. In addition, when we added generic conspiracy beliefs, religiosity, and ideology as covariates to this moderation model, neither collective narcissism nor belonging were significant predictors. On the other hand, these three variables explained changes in outgroup Covid-19 conspiracies to a significant degree. This finding is compatible with literature: Numerous studies showed close relations between religiosity, generic conspiracy beliefs, ideology and Covid-19 conspiracies (e.g., [2]).

Secondly, we hypothesized that grandiose narcissism predicts Covid-19 conspiracies with the moderating effect of ‘need for uniqueness’. This hypothesis was partly supported. Participants who showed high grandiose narcissistic traits were in need to feel unique and this led to belief in Covid-19 conspiracies. However, in the moderation model, grandiose narcissism alone did not predict Covid-19 conspiracies. Only when people have both grandiose narcissistic traits and higher need of uniqueness do they believe in Covid-19 conspiracies. In other words, when need for uniqueness is low or moderate, narcissism did not predict Covid-19 conspiracies. Although in the literature it was shown that narcissistic traits predicted conspiracy theories (e.g. [8]), the link between them was left unclear. In the present research, we found that higher need for feeling special may be the main reason behind narcissists believing in conspiracies. As Imhoff and Lamberty [25] argued, conspiracy theories are believed by a minority and for this reason people may feel ‘unique’ or ‘special’ by holding them. They think of themselves as enlightened because they created alternative explanations and see beyond official ones [13]. When we added general conspiracy beliefs and religiosity as covariates to this model, prediction percentage increased dramatically.

## Potential limitations and suggestions for future research

Although the current study revealed important dynamics between narcissism types and conspiracy theories, it has several limitations. Firstly, it employed cross-sectional data which cannot reveal causal relations between variables. Secondly, because Covid-19 is a comparatively new phenomenon, there were no validated measures of conspiracies related to it. Therefore, we collected the most common Covid-19 conspiracy beliefs via a non-systematic search through the social media. Similarly, we measured national identification with a one-item belongingness question rather than with one of the validated national identity scales (e.g., [28]). Another limitation of our research is that the data were collected from psychology students who were mostly female and young. Therefore, it is not representative of the Turkish population at large. Future research should attempt to replicate our results in a wider sample to increase generalizability.

We found that both narcissistic characteristics predict Covid-19 conspiracies. However, believing in a conspiracy theory and disseminating it are importantly different. In the current research, we focused only on the endorsement aspect of conspiracy theories. Regarding dissemination, collective and grandiose narcissism may follow different patterns. Because narcissistic traits are related to the desire of being the center of attention, disseminating conspiracy theories may satisfy this need for grandiose narcissists. On the other hand, for collective narcissism, protecting and aggrandizing own nationality while putting the blame on others would be the motivation to propagate conspiracy theories among the public. Consistent with this view, Sternisko and her colleagues [33] collected data from 56 countries and found a consistent relationship between collective narcissism and believing and spreading Covid-19 conspiracies. However, while we were writing this paper, there were no studies focusing on grandiose narcissism and spreading Covid-19 conspiracy theories. Future studies may examine the relationship between narcissism and spreading Covid-19 conspiracies.

## Conclusion

Belonging to a group and feeling unique are two common needs shared by most people [25]. We hypothesized that these needs can explain the links between two narcissism types (collective and grandiose) and belief in Covid-19 conspiracy theories. Our hypotheses were partly supported; the link between grandiose narcissism and Covid-19 conspiracies was moderated by the need to feel unique. On the other hand, our expectation about the moderating effect of belonging to a national group on collective narcissism and Covid-19 conspiracies was not supported. It is possible that there are other factors which push collective narcissists to embrace these theories, which future studies should look into.

## Data Availability

The datasets generated during and/or analysed during the current study are available from the corresponding author on reasonable request.

## Appendix

1. The Corona Virus was prepared in laboratories in China and was released to the public intentionally.
2. Scientists and doctors in Turkey exaggerate the seriousness of the disease because they receive money from pharmaceutical industries.
3. The current pandemic is actually a kind of flu pandemic while the ‘coronavirus’ is something big pharmaceutical industries make up for profit.
4. The state aims to scare the public by showing the number of patients higher than it actually is.
5. With the coronavirus vaccine, the intention is to change people’s DNA.
6. The media is trying to make the state look bad by exaggerating the coronavirus disease.
7. The government deliberately hides the harmful effects of the coronavirus vaccines from the public.
8. Bill Gates aims to implant microchips in people's bodies through vaccines.
9. The state is trying to dominate the people by making restrictions on the coronavirus as an excuse.
10. The coronavirus was created by certain groups in order to reduce the world population by creating a global impact.
11. The government aims to follow people by barcoding, using the epidemic as an excuse.
